# The Potential Risk of *Bactrocera dorsalis* (Tephritidae) Invasion into the Fruit Industry in the Iberian Peninsula: A Review

**DOI:** 10.3390/insects16090969

**Published:** 2025-09-16

**Authors:** Chandana Dammika Wijekoon, Amílcar Duarte, Luís Neto

**Affiliations:** 1 Department of Zoology, Faculty of Science, University of Ruhuna, Matara 81000, Sri Lanka; chandanadammika1984@gmail.com; 2 MED—Mediterranean Institute for Agriculture, Environment and Development, CHANGE—Global Change and Sustainability Institute, Faculdade de Ciências e Tecnologia, Universidade do Algarve, Campus de Gambelas, 8005-139 Faro, Portugal; lneto@ualg.pt

**Keywords:** fruit flies, fruit production, invasive species

## Abstract

*Bactrocera dorsalis* (Hendel, 1912) (Diptera: Tephritidae) is considered a highly invasive fruit fly species already widespread in Indo-Asia and sub-Saharan Africa. In recent years, this species has extended its range into new regions previously considered unsuitable, posing an alarming new risk for the subtropical regions of Europe. This review aimed to assess the potential risk of *B. dorsalis* invasion into the fruit industry in the Iberian Peninsula, the southwesternmost tip of Europe. With the collected information from published scientific knowledge on *B. dorsalis*’s distribution, biology, climatic tolerance, host plant diversity, and recent invasion pathways, we have provided an assessment of the possibilities of introducing this alarming fruit fly species to the Iberian Peninsula. In this review, we found that *B. dorsalis* has been recently reported in several adjacent European countries such as France, Italy, and Austria, and it serves as a warning signal to the Iberian Peninsula for the possible risks that this species poses in the future. Considering this persistent threat posed by *B. dorsalis*, we reviewed its potential future threat by considering the status of the fruit industry and Mediterranean climate in the Iberian Peninsula. We also considered the findings of recent climatic forecasting models. This review provides a theoretical basis for the prospective intrusion of *B. dorsalis* into the Iberian Peninsula’s fruit industry, as well as the potential impact following its establishment, together with recommendations for restricting the introduction and spread.

## Simple Summary

The Oriental fruit fly (*Bactrocera dorsalis*) completes its larval development within the fruits of various plant species. Infested fruits become unsuitable for consumption, resulting in significant economic losses. To mitigate these infestations, farmers must apply phytosanitary products, which, in addition to the economic costs, contribute to environmental degradation. This pest has successfully spread across most countries in South Asia and sub-Saharan Africa. Although isolated introductions have occurred in parts of Europe and the Americas, *B. dorsalis* has not yet established permanent populations in these continents. This article evaluates the potential risk of B. dorsalis introduction into the Iberian Peninsula. We conclude that there is a serious risk of *B. dorsalis* being introduced into the Iberian Peninsula, which could lead to considerable economic, social, and environmental consequences for both Spain and Portugal.

## 1. Introduction

*Bactrocera dorsalis* (Diptera: Tephritidae) is one of the five most serious and alarming fruit fly pests in the world [1,2,3]. It is highly polyphagous and is regarded as one of the world’s top pest invaders [4]. Compared to the other *Bactrocera* fruit flies, *B. dorsalis* typically maintains a high pest status [5], resulting in a substantial economic loss through direct fruit damage [6]. Due to causing significant fruit damage, *B. dorsalis* is considered an A1 quarantine pest by the EPPO (European and Mediterranean Plant Protection Organization) and CABI [6,7], highlighting the need to prevent its spread through the global fruit trade.

*B. dorsalis* was first recorded in only a few countries from 1910 to 1990, but in the past three decades, it has been detected in many new habitats in 70 more countries, including European countries, indicating its rapid spreading ability [8]. This pest can invade new habitats and establish rapidly due to its high dispersal and adaptation capacity [9]. This widespread ability of *B. dorsalis* is explained by Leblanc [10] and Nugnes et al. [2] by the fact that *B. dorsalis* has high polyphagy and a high reproduction rate, as well as adaptative strategies to survive throughout the year.

For the reasons indicated above, fruit production areas that have a climate suitable for this insect and that simultaneously have a diversity of potential hosts throughout the year will have a high risk of developing important populations of this pest if the insect enters the region.

The Iberian Peninsula is located at the southwestern tip of Europe. It is Europe’s second-largest peninsula, covering around 583,256 km^2^ and accounting for approximately 5.9% of the continent. The Iberian Peninsula is occupied by Spain (492,175 km^2^) and Portugal (89,015 km^2^). The two major climate types that characterize this peninsula are oceanic and Mediterranean. The oceanic climate prevails along the Atlantic coast, with chilly summers and a mean temperature around 0 °C in the coldest month. Most of Iberia has a Mediterranean climate, with mild, rainy winters and hot, dry summers, but with very variable temperature and precipitation levels. This climate is extremely beneficial to the development of many fruit crops.

For the reasons mentioned above, Spain and Portugal are long-time fruit producers, mainly in the southern regions of both countries, and Spain is famous as the main European producer of tropical and subtropical fruits [11,12,13].

According to the FAO [14], Spain holds 25.1% of Europe’s fruit cultivation. Its contribution to European production in 2022 was the following: bananas (56.9%), cherries (18.9%), watermelon (43.4%), lemons (59.0%), berries (34.7%), avocados (71.6%), and other tropical fruits (56.1%) [14]. Portugal had an 8% fruit production share in Europe in 2022, according to the Agricultural statistics [14]. Further, in Portugal there are 259,670 hectares of fruit harvesting lands according to the FAO [14]. The share of country’s fruit production to the Europe in 2022 was the following: grapes (5.7%), apples (2.9%), oranges (6.2%), pears (10.59%), cherries (5.0%), avocados (12.7%), bananas (5.5%), berries (8.9%), and other tropical fruits (7%) [14]. In recent years, fruit growing in the south of the Iberian Peninsula has developed through the growth of the area under avocado [15] and raspberry cultivation, but also through the introduction of new fruit crops such as dragon fruit [16]. Fruit production is therefore an important sector for the Spanish and Portuguese economies, but also for the European Union economy and for Europe’s fruit supply.

In addition to being fruit producers, these countries are also large fruit consumers, which is associated with the Mediterranean diet practiced in these countries, which includes the consumption of many fruits [13]. Therefore, they are also major fruit importers. To meet consumer demand, both countries import fruits from non-European countries. Over the past years, Spain has become the fifth-largest importer of fresh fruits from non-European countries, with imports worth EUR 1.9 billion [17].

Fruit production in the Iberian Peninsula is hampered by the presence of numerous pests [18,19,20,21,22]. Many of them were imported from other continents, such as the fruit fly *Ceratits capitata*, which is currently the most important pest of most fruit crops in this region [23]. In recent years, new pests have arrived in the Iberian Peninsula, such as the African citrus psylla, capable of establishing itself in most citrus cultivars grown in Spain and Portugal, but which has had difficulty adapting to the climatic conditions of most of the citrus-producing regions of these countries [21,22,24,25,26]. This is a rare case where, although this pest has entered the Iberian Peninsula, it is currently in regression and is considered eradicated from some of the invaded areas [22].

The purpose of this paper was thus to provide an outlook on the potential risk of *B. dorsalis* invasion into the Iberian Peninsula (Spain and Portugal) by reviewing the existing literature on *B. dorsalis*’s distribution, biology, climatic tolerance, host plant diversity, and recent invasion pathways, and further to discuss possible ways of invasion and impacts of its establishment. This knowledge will be useful to researchers, quarantine officials, fruit industry experts, and agronomists studying this alarming fruit fly species or dealing with fruit import from countries where this pest is established.

## 2. Origin and Current Distribution of *B. dorsalis*

The origin of *B. dorsalis* is placed in the tropical region of South-East China [27] in 1912, but was first identified in Taiwan, and only later, in 1934, was reported in mainland China [28]. However, it seems that the insect was already spread throughout the Asian region before the beginning of the 20th century [29]. Over the past century, it has been detected in many South- and Indo-Asian countries, including Sri Lanka [30], Pakistan, India, Bangladesh, Nepal, Bhutan, Myanmar, China (including Taiwan), Hong Kong, Thailand, Vietnam, Cambodia, Laos, Malaysia, Singapore, the Philippines, and Indonesia [9]. Most of China’s regions have reported the invasion of this species [31]. According to Mutamiswa et al. [32], during the 20th and 21st centuries, the distribution pattern of *B. dorsalis* has not changed considerably in South Asia. Some areas occupied by the insect were later considered eradicated, mainly on islands, but also in small continental regions of Australia [33].

Since the middle of the 20th century, *B. dorsalis* has invaded continents other than Indo-Asia, extending its range of distribution. The Northern Mariana Islands (1935) reported the first incidence outside of Asia, followed by Hawaii (1945) and Guam (1947) [34]. It was initially identified in Suriname, South America, in 1975 [8], and was annually detected in California and Florida, USA [35]. In 2003, this species was reported for the first time in Africa (Kenya) [36], and it has since established itself in all sub-Saharan Africa [37].

There have also been reports of *B. dorsalis* from Europe in recent years. Italy’s Campania region (2018, 2019) was the site of the first known report of *B. dorsalis* in Europe [2]. Specimens of *B. dorsalis* were found in Vienna, Austria [34,38,39]. In the period 2019 to 2023, several individuals of *B. dorsalis* were also captured in southern France, but it is not considered established in this country [40,41].

## 3. Biology of *B. dorsalis*

### 3.1. Life Cycle

The life cycle of *B. dorsalis* includes eggs, three larval instars, pupae, and adults. Adult fruit flies emerge about 14 days after egg-laying under tropical temperature conditions [42]. Flying gravid females select their host fruit and lay eggs into it by piercing the fruit peel with the ovipositor. During her life, in optimal conditions (T = 28 °C), a mature female can lay approximately 3000 eggs [32]. Under field conditions, a female lays between 1200 and 1500 eggs [43,44], with a hatching success of approximately 85%, depending on the environmental conditions. They often lay eggs in clusters of 3–30 in the flesh of the host fruit. After 1–2 days, the eggs hatch and the larvae begin to feed on the fruit’s flesh. The larvae of *B. dorsalis* are elongated and have a typical cylindrical-maggot shape [32]. The larva undergoes three phases (sheds twice) of development, with each stage being developed by feeding on the fruit pulp. Then, the larva makes a hole to exit the fruit 10 days after the egg hatches [34], falls to the ground, buries itself, and pupates. Pupae are barrel-shaped and dwell in the soil. However, mature larvae that are unable to leave the fruit in time can form pupae inside it [31]. Adult flies emerge after 4–6 days of pupation [42].

Adults are slightly larger than house flies when they first emerge, and males and females are nearly identical in size [32]. All recognizing morphological characteristics are fully developed seven days after emergence [45]. A thin ovipositor distinguishes mature females. After 6–12 days of emergence, adults of both sexes reach sexual maturity [32,34]. *Bactrocera dorsalis* shows multiple overlapping generations per year [34].

### 3.2. Climatic Conditions for B. dorsalis

*Bactrocera dorsalis* is a tropical fruit fly. Temperature, relative humidity, and rainfall all have a significant impact on the survival, flight behavior, and population dynamics of this insect [46]. Temperature is the most important determinant among them [47,48], and so tropical climates with steady temperatures throughout the year fulfill the needs of the *B. dorsalis* establishment. At low temperatures (<16.7 °C), females are not able to lay eggs, but their longevity increases, being able to live up to 117 days (at 18.8 °C) [49]. The ideal temperature for *B. dorsalis* larval development is between 25 °C and 30 °C [41,50,51]. The critical thermal minimums (CT_min_) for adult and larval activity are 9.1 °C and 7.3 °C, respectively [52].

*Bactrocera dorsalis* was shown to be more tolerant to extreme environmental conditions than other *Bactrocera* fruit flies [3]. This suggests that *B. dorsalis* is able to survive and establish itself outside tropical regions. The optimal temperature for the development of *B. dorsalis* is 25–30 °C [31]. Despite its tropical origin, *Bactrocera dorsalis* has been shown to have survival adaptations to temperate regions [3,53]. Furthermore, immature stages could adapt and develop at varying moisture levels (10–60%) [31]. In temperate climates, *B. dorsalis* could survive in the summer if its host plants are available [54]. Furthermore, it was shown that though *B. dorsalis* is adapted to tolerate winter climates; its developmental stages take longer than average [54]. Under extreme cool conditions, *B. dorsalis* eggs, pupation, and adult life duration may be extended, respectively, up to 20 days, 90 days, and 12 months longer than their normal spending days [3,54,55]. According to the CABI and EPPO [6], *B. dorsalis* is adaptable to a variety of environmental conditions, including dry winters. The rainfall pattern and precipitation amount affect *B. dorsalis* population dynamics [31], and its abundance notably falls under high precipitation conditions [56]. Furthermore, *B. dorsalis* performs best in humid and hot environments [56]. The overall effect of environmental temperature and moisture on *B. dorsalis* development is mostly determined by vegetation type, agricultural practices, and precipitation pattern [56].

### 3.3. Host Plants

Aside from climatic influences, the population dynamics of *B. dorsalis* are influenced by host plant abundance and fruiting seasons [31,50,57,58]. The abundance and diversity of host plants change depending on environmental conditions. Most studies have demonstrated that there is clear variation in the *B. dorsalis* population with the seasons of fruit harvesting [59]. *Bactrocera dorsalis* is an extraordinarily polyphagous fruit fly species, with more than 300 host plants recorded [51]. The range of known host plants for this species has increased since 1960; in 1960, 150 host plant species were reported; in 2003–2005, 250 host plant species were recorded [31,60], but currently more than 600 host plants worldwide are known [60,61]. The selected common host plants of *B. dorsalis* are mentioned in Table 1.

Most *B. dorsalis*’ host plants are commercially important in tropical agriculture. Several studies identified *Mangifera indica* (mango), *Capsicum annuum* (chili pepper), *Annona muricata* (soursop), *Coffea canephora* (robusta coffee), *Eriobotrya japonica* (loquat), and *Citrus japonica* (kumquat) as globally distributed host plants of *B. dorsalis* [60,61,66,67,68,69,70].

Many host plants cultivated in subtropical and temperate climates are commercially significant in European Union countries and particularly in the Iberian Peninsula. The main tropical host plants are important for assessing the risks of *B. dorsalis* spread in fruit packaging, import, and export. Host plants growing in all climatic conditions should be considered when identifying possible risks for *B. dorsalis* invasion and establishment [34]. The most important element in keeping a viable population of *B. dorsalis* in tropical countries is an ongoing fruit supply. Fruit flies are assured a constant supply of fresh fruits even in temperate locations by the major fruits for a certain period, and the availability of alternative host fruit species afterward [60].

## 4. The Trend of *B. dorsalis*’s Invasion of the European Region

The study of Zeng et al. [8] showed that *B. dorsalis* has increased its invasion dramatically since 1990, reaching 111 (89.5%) regions at a speed of 4.11 per year. Further, from 1991 to 2017, *B. dorsalis* has shown a south-western expansion from China to tropical and subtropical regions of Oceania and Asia, and in the 2001–2010 period, it invaded an average of 4.7 regions per year in Asia and Africa. Remarkably, during the last few years, *B. dorsalis* expanded its distribution ranges to other countries or regions in Africa and some places in Europe [8]. This westward-oriented invasive pathway of *B. dorsalis* is well addressed [31,71]. In 2007, CLIMEXTM was used to predict the invasion pathway of *B. dorsalis* under current and future climatic changes, and this study predicted its invasion trend towards the Polar Regions with the changes in global temperature [54]. This northward invasion tendency of *B. dorsalis* with future temperature increases is further shown using molecular data, and this study proved the relationship between *B. dorsalis* spreading and increasing global temperature [72]. Moreover, it was indicated that the distribution of *B. dorsalis* is continuous and that high-latitude regions will be its habitats in the future [8].

The model approaches applied by different authors [28,29,54] to estimate the *B. dorsalis* distribution revealed that the majority of its potentially suitable places are in South America, Africa, and Europe. All these studies brought attention to the potential risk of *B. dorsalis* spreading in the Mediterranean coastal areas of Europe.

Furthermore, under future climate conditions, the risk areas of *B. dorsalis* in the Northern Hemisphere will increase further northward in Europe by 2070 [28]. Dong et al. [73] demonstrated the potential of coastal locations in Western Europe for future *B. dorsalis* establishment. By 2070, the risk regions for *B. dorsalis* in Europe are anticipated to grow by more than 1,000,000 km^2^, or 12% of the land area [28]. Moreover, Qin et al. [28] predicted that by 2070, around 20% of European locations will be climatically favorable for *B. dorsalis*. There are records of recent *B. dorsalis* invasions in new regions of China that were previously thought to be critically unsuitable for its survival due to overwintering cold stress [28,54], but these areas have similar climatic characteristics to temperate regions of Europe [51]. It is predicted that *B. dorsalis* could eventually acclimatize to Europe. The distribution of *B. dorsalis* in Europe is assumed to be northward across much of Spain, Italy, southern France, and Portugal [54,73]. Furthermore, Croatia, Slovenia, Greece, and Cyprus, as Mediterranean coastal areas, were proposed, as well as parts of France’s Atlantic coast, as future possible risk zones in Europe [51].

*B. dorsalis*’ natural distribution and invasion pathway are inextricably related to global temperature fluctuations. In addition to the global temperature scenario, increased worldwide trade in fruits and vegetables is another probable avenue for *B. dorsalis* to spread rapidly [8,9]. Furthermore, host availability, interspecific competition, and the impact of natural enemies are energetic factors that influence *B. dorsalis* potential distributions [8]. According to Baker et al. [74], the fruit availability in the winter months of some temperate countries increases the distribution range of this fruit fly species. The host plants of *B. dorsalis* expanded towards higher latitudes and northern temperate zones in tandem with the rise in global temperatures [9]. The most significant host plant for *B. dorsalis* in the tropics is the mango [60]. De Villiers et al. [51] reported that rising global temperatures permit mango cultivation to spread in range and yield, which in turn encourages *B. dorsalis* colonization of temperate regions. As shown by Zeng, et al. [8], *B. dorsalis* could migrate for long distances. This unusual behavior of *B. dorsalis* was also evidently proven in Hawaii [75], where *B. dorsalis* was shown to fly about 37 km (about 22.99 mi), and similar observations were also made in Malaysia [76]. According to CABI [6], it can fly about 50–100 km. These findings raise the possibility of *B. dorsalis* invasions from neighboring nations by long-distance flying. However, these long flights appear to be rare, since the main objective of adult flies is to find fruit for oviposition [77]. Furthermore, long-distance flights are very energy-intensive [78]. Thus, in most cases, the flights of this species do not exceed a few hundred meters and long flights, capable of dispersing the pest over long distances, are exceptional [79,80].

The danger posed by *B. dorsalis* was also brought to light by several studies [38,60], which highlighted that due to the species’ possible extensive flight range, infected fruit imports or flying adults could be introduced to the neighboring countries of Italy, France, and Austria, which do not currently have it. Zarpas et al. [63], reported that the importation of tropical and subtropical fresh fruits from areas where the pest has already established itself is the most important pathway for the introduction of non-EU fruit flies into Europe. Additionally, when travelers carry fruit in their luggage, they can disperse *B. dorsalis* larvae [2].

The EPPO [60] assessed the presence of *B. dorsalis* in the EPPO regions as ‘minimal’ and ‘transient’. Whereas, under future climate change scenarios, there is a significant possibility of *B. dorsalis* invasion and establishment in EPPO countries, as indicated by subsequent investigations. As a result, the EPPO should consider updating the assessment categories about *B. dorsalis*’ potential pest danger.

## 5. The Invasion Risk of *B. dorsalis* to the Fruit Industry in the Iberian Peninsula

Although the presence of *B. dorsalis* has already been documented in adjacent France, Italy, and Austria, it is imperative to consider the potential risk of introducing *B. dorsalis* to the Iberian Peninsula’s fruit sector. The identification of *B. dorsalis* in France, Italy, and Austria serves as a warning that this dangerous fruit fly is making conquests into other European countries. Dong et al. [73] unequivocally demonstrated that *B. dorsalis*’s impending invasion of Europe poses a major threat to four significant temperate fruits: oranges, pears, apples, and peaches. The Iberian Peninsula cultivates these fruits and imports them from both developed and developing countries. Furthermore, the main host fruits of *Bactrocera* fruit flies in Europe will be *Citrus* spp., *Prunus* spp., *Persea americana*, and *Mangifera indica* (mango) [34]. Although the oviposition signs can be observed in fruits, when the attacks are very recent, these signs go easily overlooked. Therefore, implementing effective post-harvest and transport management practices is currently the only reliable strategy to prevent the spread of live insects to new areas.

According to the EPPO and Clarke et al. [81,82], the constant fruit supply necessary for the potential establishment of *B. dorsalis* in Europe is ensured by the availability of citrus from September to June and the following presence of many other host fruits from May to September. Additionally, *B. dorsalis* has been intercepted multiple times entering Europe from countries that had previously reported infested crops, mostly mangoes [7,73]. Notably, *B. dorsalis* was shown to have a host shift pattern that included pear (*Pyrus communis*), jujube (*Zizyphus jujuba*), persimmon (*Diospyros kaki*), and sweet orange (*Citrus ×sinensis*), as well as pupal survival, even at very low soil temperatures [53].

Remarkably, most tropical and subtropical fruits are cultivated in Spain, including bananas, avocados, mangos, cherimoyas, papayas, pineapple, and carambola, and the introduction of additional tropical fruits such as pitayas, passion fruit, mamey, guava, and litchi is taking place [12,16,83]. In the same way, southern Portugal also engages in small-scale cultivation of many of these fruit plants [60]. The cultivation of tropical and subtropical fruits, which can be found in Europe’s Mediterranean regions, including Spain and Portugal, has been reported by Zarpas et al. [63] as posing a risk to the establishment and spread of non-EU Tephritidae fruit flies. Further, it has been emphasized that many countries in Mediterranean Europe provide the uninterrupted availability of suitable host fruits for non-EU fruit flies, and even in winters, they could survive with a slowly growing population rate [63].

The availability of even one suitable host could lead to the establishment and spread of non-EU fruit flies. As a result, it is obvious that the Iberian Peninsula is at great risk of being infested by dangerous Tephritidae fruit flies [63], including *B. dorsalis*. Therefore, there is a high probability that *B. dorsalis* will be introduced into the Iberian Peninsula fruit industry.

Considering all the above-mentioned factors, there is a high chance of *B. dorsalis* invasion and establishment in the fruit industry in the Iberian Peninsula. Within areas with a Mediterranean climate, due to the low frost probability, it is the southern coastal area that has the highest concentration and variety of fruit trees and the one that is most suitable for the development of tropical and subtropical fruit trees. It will therefore be in this area where the probability of developing important populations of flies will be greater. However, the forecast for the coming decades is for an increase in the average, maximum, and minimum temperature for the entire Iberian Peninsula [84], naturally expanding the area with fruit aptness and consequently the area with good conditions for the development of flies.

## 6. The Possible Impact of *B. dorsalis* Establishment in the Iberian Peninsula

Since there are no successful agricultural management strategies, excluding pesticides, to contain the spread of this insect in a suitable area, after an introduction of *B. dorsalis*, the only factors that will limit its establishment are the presence of hosts and climatic factors. While mulching has been shown to increase soil organic matter [85] and may influence pupal survival [86], soil management practices alone are insufficient to effectively control this pest. Soil moisture also plays a key role in pupal survival and adult emergence [87,88], and heavy rainfall can lead to high pupal mortality. However, pest control through soil moisture regulation appears impractical under field conditions. Other soil physical characteristics, although they can influence pupal mortality, are not limiting factors for insect development. Thus, the accidental introduction of *B. dorsalis* into the Iberian Peninsula fruit sector should be cause for concern, as the country’s climatic conditions and host plant diversity will be conducive for its survival and development.

In the case of Faro, located in southern Portugal, climatological normals from 1971–2000 indicate an average minimum temperature of 7.3 °C in the coldest month (January) and an average maximum of 29.0 °C in the hottest month (July). During this period, the absolute minimum and maximum temperatures recorded were −1.4 °C and 39.8 °C, respectively [89]. Although extreme temperatures may cause some mortality in exposed individuals, *B. dorsalis* is likely to survive in sheltered microhabitats within the tree canopy, where conditions are more moderate.

Similarly, in Málaga, southern Spain, data from the 1981–2010 climatological normals [90] show an average minimum temperature of 7.4 °C in January and an average maximum of 30.8 °C in July. The average number of frost days per year is only 0.2. This region hosts the highest concentration of tropical and subtropical fruit crops in Spain, providing abundant resources for B. dorsalis. As in Faro, the combination of favorable climatic conditions and host plant diversity suggests a high likelihood of survival and population establishment in this area.

*Bactrocera dorsalis* is a superior competitor, and its invasion of new environments has resulted in the competitive displacement of previously established fruit fly species.

In La Réunion and in French Polynesia, *B. dorsalis* displaced dominant fruit flies that were already present there [27,41,91]. In Africa, *B. dorsalis* competitively replaced four established fruit fly species: *Ceratitis rosa*, *C. cosyra*, *C. quilicii*, and *C. capitata* [41,50,92]. *Bactrocera* fruit flies are competitively superior to *Ceratitis* species [93], especially in low-lying areas, just above sea level [94]. The Mediterranean fruit fly, *C. capitata*, is well established as a first rank economically significant fruit fly species in the Iberian Peninsula [95]. Many of the host fruits overlap between the two species, but *B. dorsalis* has a wider host range [96]. Biasazin et al. [97] recorded the displacement of the previously dominating Mediterranean fruit fly in Ethiopia following the invasion of *B. dorsalis*. Furthermore, the displacement of additional *Bactrocera* spp. (*B. tryoni*, *B. kirki*, and *B. perfusca*) has been documented in French Polynesia because of *B. dorsalis* incursion [45,54]. As a result, an intrusion of *B. dorsalis* into the Iberian Peninsula could have a significant impact on the country’s current *C. capitata* population.

Moreover, the Iberian Peninsula imports fruits from Africa, India, and China, where *B. dorsalis* is already widely distributed. According to Ullah et al. [58], *B. dorsalis* is estimated to have caused an economic loss of citrus in China of between USD 10.037 and 45.584 billion. Dong et al. [73] also reported that *B. dorsalis* damage to apple, peach, and pear production in China is substantial.

There are several negative economic impacts that arise with the introduction of *B. dorsalis* to the Iberian Peninsula. It will severely affect the volume of fruit production from potential hosts cultivated in the Iberian Peninsula. Additional phytosanitary treatments will be needed to control *B. dorsalis*. In this case, the Iberian Peninsula has to base itself on what is conducted in already infested countries that have similar climate conditions. Indeed, these treatments have an economic cost, environmental impact, and potential risk to the health of consumers. Further, there will be additional limitations on the export of fruit from the Iberian Peninsula to countries where *B. dorsalis* is a quarantine pest. Currently, the presence of *C. capitata* in the Peninsula limits exports to some countries (the USA and Canada, for example). Other environmental impacts would mainly be the decrease in biodiversity in fruit orchards resulting from a greater number of treatments. In addition, the EU would be faced with a dilemma: authorize more treatments or stop producing fruits from the most sustainable species.

Furthermore, due to its high reproduction ability, *B. dorsalis* might spread and settle swiftly after intrusion, and Nugnes et al. [2] showed that *B. dorsalis* would be the most serious threat to European fruit orchards in the future.

## 7. Recommendations for Preventing the Introduction

In this article, we have given a general idea, based on the bibliography, about which entry routes carry the highest risk. We can rely on information about imported fruits that are hosts for *B. dorsalis* and that come from countries infested by this fly. According to Nugnes et al. [2], airports and ship ports are high-risk viable routes for *B. dorsalis* entering Europe. More studies should be carried out regularly on the subject to detect the future invasion of *B. dorsalis.*

It is recommended that a risk assessment be conducted to determine the potential risk factors for *B. dorsalis* invasion and establishment in the Iberian Peninsula’s fruit industry. The prospective invasion routes, host plants that are now present and their fruiting phenology, and more climatically favorable regions of the country should be the main emphasis of this survey. Additionally, the EPPO [60] recommended that every member state conduct this risk-based survey. Furthermore, Taddei et al. [98] highlighted the necessity for such implementations in Europe; quick and accurate identification by European plant health authorities is critical for avoiding its importation and establishment. Furthermore, custom phytosanitary services at airports needs improvement to detect any infestations in passenger luggage, as Nugnes et al. [2] stated that this would be a viable pathway for *B. dorsalis* to enter Italy. Furthermore, continuous random methyl-eugenol fruit fly traps in fruit orchards could be utilized to detect the presence of *B. dorsalis* in the Iberian Peninsula.

## 8. Conclusions

Focusing on the risk analysis of *B. dorsalis* invasion, we summarize and provide an outlook that the Iberian Peninsula’s fruit industry is at high risk of *B. dorsalis* incursion due to host fruit availability and climatic preference. A thorough risk assessment survey, as well as upgrades of the early detection systems at potential entrance sites into the two countries, such as seaports, airports, fruit packing, sorting, and processing centers, and fruit import and transit end points, are highly suggested.

## Figures and Tables

**Table 1:** Common host plants of *B. dorsalis*.

Host Plant Species	Common Name	Main Distribution ^1^	References
*Anacardium occidentale*	Cashew	Trop.	[60,62,63]
*Ananas comosus*	Pineapple	Trop., subtrop.	[60,62,63]
*Annona* spp. *	Custard apple	Trop.	[4,28,51,63,64]
*Artocarpus altilis*	Breadfruit	Trop.	[4]
*Artocarpus heterophyllus*	Jackfruit	Trop.	[4]
*Averrhoa carambola*	Star fruit	Trop.	[4,62]
*Carica papaya* *	Papaya	Trop.	[34,51,60,63]
*Chrysophyllum* spp.	Star apple	Trop.	[4,51]
*Citrus* spp. *	Orange, lemon, mandarin, tangerine, and lime	Trop., subtrop., temp.	[4,28,51,60,62,63,64]
*Coffea* spp.	Coffee	Temp., trop., subtrop.	[34,51,63]
*Cucumis melo* *	Melon	Temp., Trop., subtrop.	[4,51,60,62,63]
*Cucumis sativus* *	Cucumber	Temp., trop., subtrop.	[28,34,60]
*Diospyros kaki* *	Persimmon	Temp., trop., subtrop.	[4,62,63]
*Eriobotrya japonica*	Loquat	Temp., trop., subtrop.	[4,28,51,62]
*Ficus carica* *	Fig	Temp., trop., subtrop.	[60,63]
*Garcinia mangostana*	Mangosteen	Trop.	[34,60]
*Irvingia gabonensis*	African apple	Trop.	[64]
*Malus domestica* *	Apple	Temp., Subtrop.,	[28,34,51,60,63]
*Mangifera indica* *	Mango	Trop., subtrop.	[4,28,34,60,62,63,64]
*Manilkara zapota*	Sapodilla	Trop.	[4,51]
*Musa* spp. *	Banana	Trop.	[4,60,62,63]
*Nephelium lappaceum*	Rambutan	Trop.	[28,34,60]
*Passiflora edulis* *	Passion fruit	Trop.	[28,60,62,63]
*Persea americana* *	Avocado	Temp., trop., subtrop.	[4,62,63]
*Prunus armeniaca* *	Apricot	Temp., trop., subtrop.	[28,51,60,63]
*Prunus avium* *	Sour cherry	Temp.	[28,34,60,63]
*Prunus domestica* *	Plum	Temp., subtrop.	[28,60,63]
*Prunus persica* *	Peach and nectarine	Temp., subtrop.	[34,51,60,63]
*Psidium guajava*	Guava	Trop.	[4,28,51,60,63]
*Punica granatum* *	Pomegranate	Temp., trop., subtrop.	[34,60,62]
*Pyrus* spp. *	Pear	Temp., subtrop.	[4,28,63]
*Selenicereus undatus* *	Dragon fruit	Trop., subtrop.	[65]
*Solanum lycopersicum* *	Tomato	Temp., trop., subtrop.	[4,60,62,63]
*Solanum melongena* *	Eggplant	Temp., trop., subtrop.	[28,51,60]
*Spondias mombin*	Yellow mombin	Trop.	[64]
*Syzygium jambos*	Rose apple	Temp., trop., subtrop.	[4,34,63]
*Uvaria chamae*	Bush banana	Trop.	[64]
*Vitis vinifera* *	Grapes	Temp., trop., subtrop.	[4,34,51,62]
*Xylotheca kraussiana*	African dogrose	Trop.	[32]

* Species cultivated in the Iberian Peninsula with economic importance; ^1^ Temp.—temperate; trop. —tropical; subtrop.—subtropical.
